# A Tool for Multiple Targeted Genome Deletions that Is Precise, Scar-Free, and Suitable for Automation

**DOI:** 10.1371/journal.pone.0142494

**Published:** 2015-12-02

**Authors:** Wayne Aubrey, Michael C. Riley, Michael Young, Ross D. King, Stephen G. Oliver, Amanda Clare

**Affiliations:** 1 Department of Computer Science, Aberystwyth University, Aberystwyth, SY23 3DB, United Kingdom; 2 Institute of Biological, Environmental and Rural Sciences, Aberystwyth University, Aberystwyth, SY23 3DD, United Kingdom; 3 Manchester Institute of Biotechnology and School of Computer Science, University of Manchester, Manchester, M1 7DN, United Kingdom; 4 Cambridge Systems Biology Centre and Department of Biochemistry, University of Cambridge, Sanger Building, 80 Tennis Court Road, Cambridge CB2 1GA, United Kingdom; Tulane University Health Sciences Center, UNITED STATES

## Abstract

Many advances in synthetic biology require the removal of a large number of genomic elements from a genome. Most existing deletion methods leave behind markers, and as there are a limited number of markers, such methods can only be applied a fixed number of times. Deletion methods that recycle markers generally are either imprecise (remove untargeted sequences), or leave scar sequences which can cause genome instability and rearrangements. No existing marker recycling method is automation-friendly. We have developed a novel openly available deletion tool that consists of: 1) a method for deleting genomic elements that can be repeatedly used without limit, is precise, scar-free, and suitable for automation; and 2) software to design the method’s primers. Our tool is sequence agnostic and could be used to delete large numbers of coding sequences, promoter regions, transcription factor binding sites, terminators, etc in a single genome. We have validated our tool on the deletion of non-essential open reading frames (ORFs) from *S. cerevisiae*. The tool is applicable to arbitrary genomes, and we provide primer sequences for the deletion of: 90% of the ORFs from the *S. cerevisiae* genome, 88% of the ORFs from *S. pombe* genome, and 85% of the ORFs from the *L. lactis* genome.

## Introduction

Techniques for the deletion of multiple genes and other genomic regions are now a standard part of the modern biologist’s toolbox [1–6]. For example multiple genes must be removed from an organism to abolish traits controlled by several functionally overlapping genes [7–10], or to engineer strains that lack several unwanted functions [11]. There are many genome-wide mutagenesis methods which alter genomes by integrating transposable elements or synthetic oligonucleotides into cells [12–14]. However, the alterations they make are irrational and these methods do not permit the targeted removal of genomic regions. Other existing deletion methods are limited in that they are designed for the removal of only a few genomic elements. This is a problem as many advances in systems and synthetic biology require the targeted removal from a genome of a large number of genomic elements. The deletion of multiple genomic elements requires marker recycling but existing methods leave behind “scars” which lead to genome instability [1, 15–20].

Other methods based on synthetic oligonucleotides have been developed to overcome this undesirable feature. However they have other drawbacks. The seamless gene deletion approach developed by Akada *et al.* [21] is *imprecise*, in that it removes a 20 bp intergenic DNA segment downstream from each deleted gene. Besides being inelegant, this could be detrimental as the removed sequences might affect the expression of the adjacent gene(s) [22]. Moreover, many RNA molecules are transcribed from highly conserved intergenic regions; they have important but as yet poorly understood regulatory functions [23, 24]. The “delitto perfetto” method developed by Storici and Resnik [25] is a versatile technique for the deletion of genes and site-directed mutagenesis in *S. cerevisiae*. It requires two transformation steps: one for the insertion of a counter-selectable *core* cassette within the target gene and another for its recovery through recombination with pairs of overlapping integrative recombinant oligonucleotides (IROs). The IROs used require *in vitro* PCR extension to increase the overlapping complementarity needed for integration. However, for reliable recombination large amounts of IROs are required, (up to 5nmol of each IRO), 500 times more primer than is used in a conventional PCR. IROs also require DNA purification before use. This coupled with the additional transformation step restricts the method’s suitablity for automation. Indeed, no existing marker recycling method is suitable for automation, and automation is an absolute prerequisite for large-scale genome engineering projects.

## Materials and Methods

### The deletion method

We took as our starting point the method of Akada *et al.,* [21], as we considered it to be the existing method closest to meeting our design criteria. The key idea of Akada et al. was to identify a 40 bp sequence in the genome, and insert it flanking the marker, generating a direct repeat after transformation, so that pop-out recombination between the repeats could remove the marker without leaving a scar. Adaka’s method was modified in a number of ways to allow the automation of the method and to allow excision of only the target sequence.

**Fig 1:**
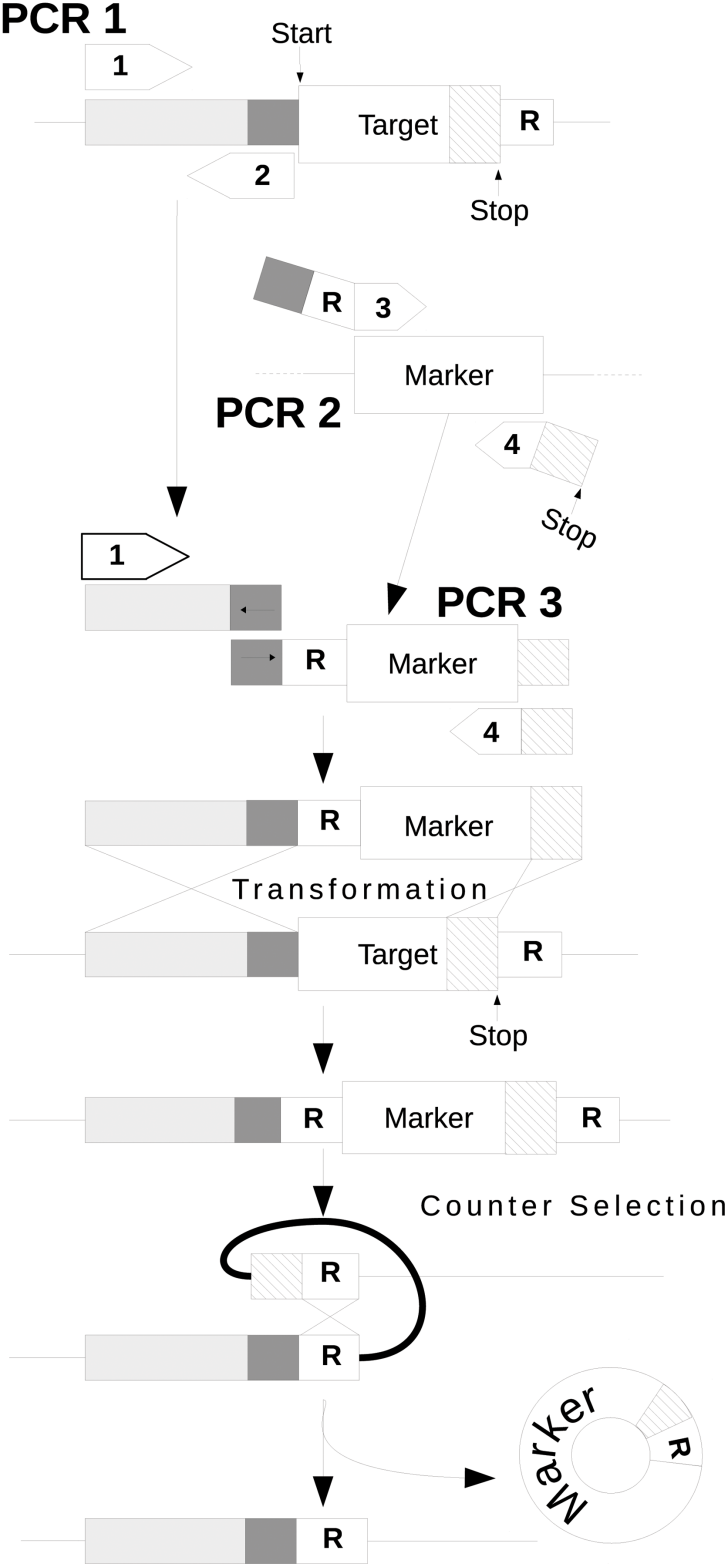
The seamless gene deletion method. Three PCR amplifications use two sets of primers that have complex dependencies. The PCR1 primers (1 and 2) are chosen so that the amplicon generated is a 500–1000 bp segment immediately adjacent to and upstream from the target gene. PCR2 generates target gene-specific variants of the marker gene(s) for fusion to the product of PCR1 and a 40 bp repeat sequence (R) copied from a region immediately downstream of the target sequence. The PCR3 is a SOE-ing reaction that fuses the products of PCR1 and PCR2 to generate a complete deletion cassette flanked by repeat sequences native to the target. Marker excision is induced by growing transformants on a minimal medium containing 5-fluoroorotic acid—a pyrimidine analogue. The repeat sequences recombine to cleanly excise the marker gene without the addition of extraneous sequences to the genome.

In contrast to the procedure developed by Akada *et al.,* the right flanking region of homology between the target gene and the deletion cassette is at the extreme 3’ end of the target gene—circumventing the unnecessary loss of ∼20bp downstream of the target ORF (see Fig 1). This makes the method truly seamless and removes the risk of unintentionally deleting functional sequences in compact genomes.

The method of Akada *et al.,* relies on the use of size purified DNA fragments to build a deletion cassette (Akada, personal communication). Our method was developed to be run by fully automated laboratory robotic equipment which is unable to size purify DNA. We adapted the method of Akada *et al.,* to use crude PCR1 and PCR2 products to produce the deletion cassette in PCR3. We achieved this by using strict primer design rules, which we optimised by trial and error and then used to build the primer design software. One such rule involved increasing the length of primers in PCR1 to allow for a high annealing temperature and reduced non-specific amplification. Also, primers in PCR1 and PCR2 are not used in equal amounts. This is to cause primer depletion thus removing the presence of these unwanted primers in PCR3. Where possible the overlapping region between PCR1 and PCR2 products was increased from 24 to 40 nucleotides.

SOEing (Splicing by Overlap Extension) PCR works best if equimolar quantities of purified DNA molecules are employed in the reaction. The amount of template added to PCR 2 was reduced 2000-fold to equal the number of primer binding sites in PCR1, resulting in approximately equivalent amounts of each amplicon being produced in PCR1 and 2. This adjustment in template ratio enabled both reactions to be run simultaneously, under identical conditions, to facilitate large-scale automation.

PCR1 primers were increased from 22 to 30–60 nt to increase product specificity and purity. We also employed primers 1 and 4 in 10-fold excess during PCR1 and PCR2 to deplete primers 2 and 3 (Fig 1). Residual primers 2 and 3 reduce the efficiency of the SOEing reaction during PCR3 by competing for binding to the region of overlap between the PCR1 and PCR2 products. This effectively avoids the need to purify the products of PCR1 and PCR2 before fusing them in PCR3 allowing the method to be fully automated.

### The algorithm and software for choosing primers

**Fig 2:**
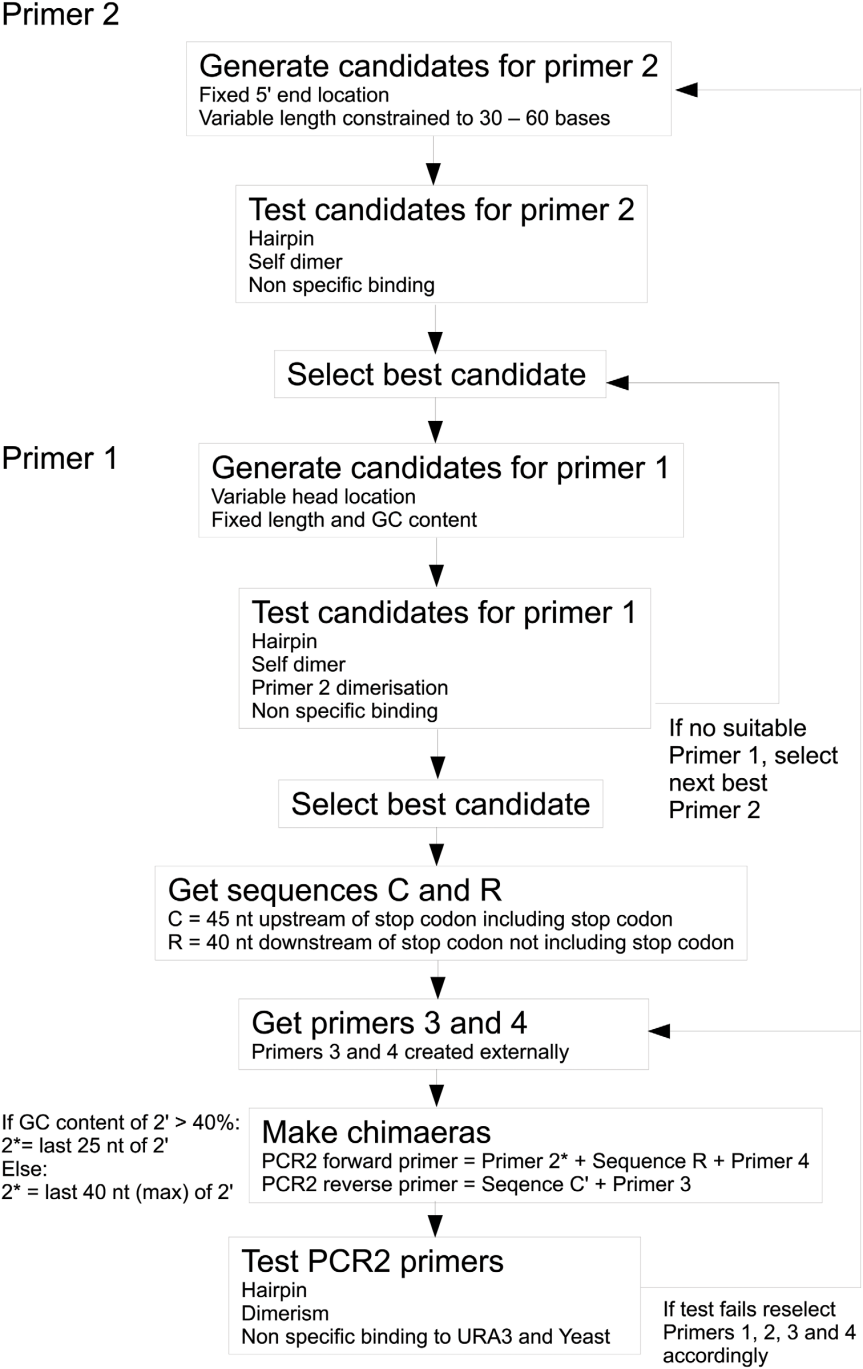
Algorithm flowchart. A flowchart depicting the algorithm for primer design, showing the order in which the primers are chosen and the tests involved in selecting successful primers.

For high-throughput work such as genome reduction, it is crucial that every step of the process is traceable, repeatable and modifiable to suit laboratory circumstances and available equipment. For this reason we publish the software used to generate the primers, so that others may reuse it, relax or tighten specific constraints and integrate it with their computational pipelines as required. The algorithm is shown in Fig 2 and important aspects are summarised below:

- The PCR1 primers (1 and 2) are chosen so that the amplicon generated is a 500–1000 bp segment immediately adjacent to and upstream from the target gene, see S1 Fig.
- PCR2 generates target gene-specific variants of the marker gene(s) for fusion to the product of PCR1. Segments at the 3’ ends of the chimeric primers (3 and 4) anneal to the marker gene template (pUG72 for *S. cerevisiae*, pFS118 for *S. pombe*), pCS1966 for *L. lactis* [26]. To avoid inclusion of the palindromic *lox*P sites that flank URA3 in pUG72, the number of potential amplicons was restricted to 4 possible pairs. Moreover, the length of this segment in primer 3 (17–20 nt) was chosen to ensure that the length of primer 3 did not exceed 100 nt.
- The efficiency of PCR3 (a SOE-ing reaction that fuses the products of PCR1 and PCR2) depends on the length and the GC content of their overlapping ends. This overlap is nominally 40 bp long but it can be increased if necessary, by extending the 5’ ends of primers 2 and/or 3 to increase their complementarity.

The Precise Deletion software was developed using the PD5 primer design software library, which is freely available as open source [27]. Table 1 includes a set of primer sequences used to delete four candidate genes.

**Table 1:** Primer sequences used to delete four candidate genes. The PCR2_F primer is chimeric and is composed of three different parts. The bold font sequence is the region that overlaps with the PCR1 product, the italicised region represents the 40 nucleotide recombination sequence (R region) and the underlined sequence is complementary to the start of the marker gene. The PCR2_R primer consists of two parts, a region that is homologous to the target region and a region that is complementary to the marker gene (underlined).

Target ORF	Primer	Primer Sequence (5’-3’)
YAL023C	PCR1 F	CGACGACAACGACGAAGGTGAGGAACTCCAACTAAAA
YAL023C	PCR1 R	GATTGCTGGACCACGGTTCGAAACAGAATGACAGTAG
YAL023C	PCR2 F	**AAGCACATACTACGCCACATAGATA** *AACCGTATATATCTTTACATAAGTACGA*
		*TATGGTATAACT* CACACATTACTTGCCTC
YAL023C	PCR2 R	TCATGCTTCTTGCTTGTCGGCAATGTCCCAAGTGGAAAACCAGTTGATCCC
		AATACAACAGAT
YBR052C	PCR1 F	CACCAGTAAGGTTCAAATGTTCGATTTAACTAACGGAAA
YBR052C	PCR1 R	TATCTATGTGGCGTAGTATGTGCTTATAATAGTGAGTAG
YBR052C	PCR2 F	**TGTTTCGAACCGTGGTCCAGCAATC** *ATTAAGATCATCGTAGTAAGTACATAAG*
		*TAAAAAACAAGA* AGCGACAAGAAGAGATAG
YBR052C	PCR2 R	TTACAAATCTTTGATACGGTCGTAAAAGGCCTTCCCTTGAAGTTGGATCCC
		AATACAACAGAT
YDL174C	PCR1 F	AGTACGGGAGACGGCTTGGACATAGATTTA
YDL174C	PCR1 R	TGTTCTTGCTTTTGTTTCCAGCTGGATCGG
YDL174C	PCR2 F	**CCAGCTGGAAACAAAAGCAAGAACA** *GTATCTGATTTTCCTTTTTCACCCTTCAC*
		*GTAAACCTGAA* CACACATTACTTGCCTC
YDL174C	PCR2 R	TCACCTGTAATCATTAGCGGGCTCGTTTGGATCAGTTTTAAAGATGATCCC
		AATACAACAGAT
YIR013C	PCR1 F	GTTTCTAACCCTCTGATGGCAAGACTTTCATCATCTTCCTGAT
YIR013C	PCR1 R	AGCGACCCTGTAATGTTATGTTTCTAGCTAGGAACAGAAAGTG
YIR013C	PCR2 F	**AGAAACATAACATTACAGGGTCGCT** *CCCATTATACTTTTTCAGCTTTCAACCTA*
		*TCGGGACAAAA* CACACATTACTTGCCTC
YIR013C	PCR2 R	TCAGAATCTGACAGTTCCGGTAAGTTCTTTGGGGATCCTTCTCTTGATCCC
		AATACAACAGAT

### Data

We used orf_genomic_1000_all.fasta (accessed on the 5th January 2010) from Saccharomyces Genome Database [28] for the *S. cerevisiae* DNA sequences, GeneDB [29] with query advice from Val Wood—19th July 2011) for *S. pombe* DNA and UCSC Microbial Genome Browser [30] (accessed on the 22nd June 2012) for *L. lactis* DNA sequences. We used pUG72 sequence AF298788.gb (accessed on the 26th November 2012) from SGD, pFS118 from addgene (accessed on the 27th July 2012) and pCS1966 from a private communication (27th June 2012, Jan Martinussen, who can supply the plasmid). Confirmation primer sequences were obtained from EuroFAN [31].

### Strains and media

The *S. cerevisiae* strain YSBN9 (Mat**a** *ura3* Δ) was used as the host for transformation experiments. The *K. lactis* URA3 gene was amplified from plasmid pUG72 [26]. Yeast cells were cultured in standard YPD medium (1% yeast extract, 2% peptone and 2% dextrose).

### PCR

We used Go *Taq* polymerase and dNTP mix (Promega) for all PCR reactions. Desalted primers (25 nmol) were purchased from Invitrogen. All primers were designed to be less than 100 nt thus removing the additional cost of HPLC purification. PCR reaction 1 used 100 pmol of primer 1, 10 pmol of primer 2 (ca. 40 nt) and ca. 0.5 *μ*g purified *S. cerevisiae* strain YSBN9 DNA as template. Both primers have perfect homology to the template and they amplify a ∼1kb region immediately up-stream of the target ORF. PCR reaction 2 used the chimeric primers, 3 and 4, with 18 bp of homology to the *URA3* cassette at their 3’ end, [26]. 10 pmol of primer 3 and 100 pmol of primer 4 was employed, together with ca. 80 pg plasmid pUG72 DNA as template. For PCR 1 and PCR 2, a Quanta Biotech thermal cycler was programmed to denature the samples by heating at 94°C for 20 s and then to perform a touchdown reaction where the annealing temperature was incrementally reduced from 65°C to 60°C for 5 cycles. After 5 cycles of (20 s at 94°C, 30 s at 65–60°C and 2 min at 68°C) amplification involved a further 25 cycles of (20 s at 94°C, 30 s at 58°C and 2 min at 68°C). PCR reactions were verified by agarose gel (1%) electrophoresis using SYBR Safe DNA gel stain from Invitrogen (cat. no S33102).

PCR 3 contained 1 *μ*l each of the products obtained in PCR 1 and PCR 2 (each ideally containing approximately 50 ng of PCR product). After 10 cycles of (94°C for 20 s, 52°C for 30 s, 68°C for 150 s), 100 pmol each of primers 1 and 4 was added. A further 20 amplification cycles followed: (94°C for 20 s, 58°C for 30 s, 68°C for 150 s), with a final extension period at 68°C for 5 min.

### S. cerevisiae transformation

Transformations were performed as described previously [32]. Transformation in microtitre plate format was performed as previously described [33].

Products from PCR1 and PCR2 were added to PCR3 in approximately equal amounts. However the amount of cassette DNA formed in PCR3 was approximated by inspection of 5 *μ*l of PCR reaction mix on a 1% agarose gel. Approximately 0.5–5 *μ*g of DNA from PCR3 was used to transform cells.

Following isolation of *URA3^+^* transformants, *URA3^−^* recombinants were counter-selected on a synthetic-complete agar medium supplemented with 100 mg/L 5-fluoroorotic acid and 50 mg/L uracil [32]. For the algorithm evaluation experiments with the 96 test ORFs the following controls were included: (a) No DNA added (negative control, 3 replicates—no colonies); (b) no cells added (sterility control, 3 replicates—no colonies); YHR163W PCR3 DNA (positive control—see above, 2 replicates—abundant colonies). The controls were plated on the same medium as the other 96 transformations. As expected from deletion cassettes with long flanking homology, several hundred colonies were obtained per *μ*g of DNA [34].

### Protocol

1. Retrieve PCR1 and PCR2 and primer sequences for target gene from S1 Primer Sequences or [44].
2. PCR1: 100 pmol of PCR1_F primer, 10 pmol PCR1_R primer, 0.5 *μ*g purified YSBN9 yeast DNA.
3. PCR2: 10 pmol of PCR2_F primer, 100 pmol PCR2_R primer, 80 pg pUG72 plasmid DNA
4. Run PCR1 and PCR2 simultaneously. Thermocycle: 94°C for 20 s, followed by a touchdown reaction from 65°C to 60°C for 5 cycles, followed by 25 cycles of 94°C for 20 s, 58°C for 30 s, 68°C for 120 s.
5. PCR 3 contained 1 *μ*l each of the products obtained in PCR 1 and PCR 2 (each ideally containing approximately 50 ng of PCR product). Thermocycle: 10 cycles of (94°C for 20 s, 52°C for 30 s, 68°C for 150 s), 100 pmol each of primers PCR1_F and PCR2_R primer was added, followed by 20 amplification cycles as follows: (94°C for 20 s, 58°C for 30 s, 68°C for 150 s), with a final extension period at 68°C for 5 min.
6. Verify PCR3 product by inspection on a 1% agarose gel and compare against calculated mass obtained from S1 Primer Sequences or [44].
7. Transform *S. cerevisiae* strain YSBN9 using 0.5–5 *μ*g of PCR3 product as described [32, 33].
8. Selected transformants on a uracil drop-out plates.
9. Retrieve colony PCR primer sequences (CPS_A, CPS_D, RinCass, FinCass) from S1 Primer Sequences or [44].
10. Verify correct fragment integration by colony PCR using target ORF—specific primers CPS_A and the universal URA3 primer RinCass.
11. Subject verified colonies to FOA selection as previously described [32].
12. Retrieve the confirmation PCR primers CPS_A and CPS_D from S1 Primer Sequences or [44].
13. Run confirmation colony PCR using CPS_A and CPS_D primers on 5FOA^*R*^ resistant colonies, and sequence PCR product. Compare sequencing results with expected sequence from S1 Primer Sequences or [44].

## Results

We have developed a deletion tool that consists of: 1) a method for deleting genomic elements that can be repeatedly used without limit and is precise, scar-free, and suitable for automation; and 2) software to design the primers necessary for the method. Our tool is sequence agnostic, and could be used to delete large numbers of coding sequences, promoter regions, transcription factor binding sites, terminators, etc from any genetically tractable organism. The method is summarised in Fig 1.

Our method is also designed to be automation-friendly. For example, the need to purify PCR products by agarose gel electrophoresis before fusing them together to generate deletion cassettes is a major limitation with respect to automation of the previously published method [21]. We have therefore refined the various PCR steps such that two “crude” PCR products can be fused together efficiently, without the need for prior purification, to produce the cassette that generates seamless, scar-free gene deletions.

### Experimental evaluation

To evaluate our tool we used it to delete precisely 107 non-essential open reading frames (ORFs) from *S. cerevisiae*. Eleven of these ORFs were initially employed as a test-bed for software development and the remaining 96 for method evaluation (S1, S2, S3, S4, S5, S6, S7 and S8 Tables. S5 Table summarises the results obtained with all 96 test ORFS including the extent to which each PCR reaction was successful and an approximation of the number of transformants obtained. For each ORF primers were designed using our software, and the method executed in accordance with the description in the Materials and Methods section.

**Fig 3:**
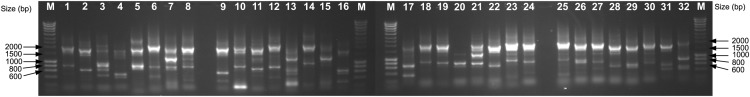
PCR3 amplifications. Example gel image for 32 of the PCR3 amplifications. Columns H contain 5 *μ*l Hyperladder 1. Expected product sizes (bp) are as follows, Rows 1–8: 2110, 1913, 2358, 1744, 1956, 2312, 2064, 2090, blank, Rows 9–16: 2080, 2159, 2041, 2332, 1833, 2235, 2163, 1811. Rows 17–24: 1862, 2152, 2084, 2079, 2024, 1918, 2121, 2062, blank. Rows 25–32: 2140, 2015, 2024, 1831, 1784, 2242, 1824, 2030. Extra bands are generally those corresponding to the sizes of PCR1 and PCR2 products. Full details given in S12 and S13 Figs, S5 and S8 Tables.

During this process, the method was refined and the limitations better understood. Fig 3 shows the amplicons obtained in PCR3 for 32 of the 107 ORFs we investigated. We verified the 11 test-bed gene knock-out strains by colony PCR using confirmation primers from the EuroFAN project. Four colony PCR products were sequenced verifying that in every case precise deletion had occurred, as expected. (S8 and S9 Figs, S1 Sequence Data and S1 Alignments).

### The software

A key step to automating multiple genomic element deletion is development of the bioinformatics algorithms to automate primer design. We developed this “Precise Deletion” software using a modular software library for primer design known as PD5 [27, 35].

The design of suitable primers for multiple genomic deletions is challenging as multiple specific primers are required for each region to to be deleted. Primer design is currently the rate-limiting step, even for small-scale projects involving the deletion of only a handful of genes.

In our deletion method primer design is additionally complicated because the three PCR amplification steps are interlinked and interdependent. For two of these steps large chimeric primers are required. In addition the DNA sequences that flank the coding sequences of some genes severely restrict the choice of suitable primers. Finally the usual *rules* need be applied to PCR primer design: similar melting temperatures, avoidance of self- and pair-dimerisation, and hairpin formation.

Our software identifies primer dimers in a novel way. As the deletion method relies on long primers, up to 100-mer, it is necessary to identify possible caterpillar-like humps in binding configurations. We identify such sequences using rule based binding affinity scoring applied only to the last 8 bases of long primers at the 3’ end [36]. We have also developed a new approach to scoring long primers for potential self- and pair-dimer formation. Among our initial test set of ORFs our software detected the potential for dimer formation in PCR2 for YGL202W and YGR125W. Therefore replacement PCR2 primers were designed for YGL202W and YGR125W using our software, and this resulted in the expected PCR2 product without apparent dimer production (S3 and S4 Figs).

Primer pairs must have similar annealing characteristics. Existing methods for the calculation of annealing temperatures do not scale well to long primers, therefore we enabled selection of primer pairs either by similar annealing temperatures, or by similar lengths and G + C contents. As the location of primer 2 in PCR1 is effectively fixed, the extent to which its overall G + C content (and to a lesser extent the G + C content at the 3’ end) can be altered is limited. It was therefore important to assess how these two factors affect the outcome. We therefore conducted experiments to investigate the range of G + C tolerance, choosing 16 ORFs to test the effect of variation in the primer G + C content overall and another 16 to test the effect of differing the proportion of G + C residues in the 8 bases at the 3’ extremity, in PCR1. The results are presented in S6 and S7 Tables and show that in general, reactions involving primers with an overall G + C content below 30% either gave a weak product or no visible product at all.

Our software performs exact local area searches in primer selection to detect secondary primer binding sites which, if present, could result in the exponential amplification of non-target DNA. This method is both more accurate and faster than applying sequence homology methods such as BLAST or FASTA. During software development we originally called BLAST and FASTA to identify potential mis-priming regions. However, PCR results indicated that two potential priming sites had not been identified and for this reason an exact local area search was developed instead. We compared our method with BLAST & FASTA and for several example ORFs in our test-set they failed to identify problematic regions of non-specific primer/template binding. The PCR1 primers for YAL065C preferentially amplified a smaller product. Sequencing revealed that primer 1 had bound to an almost identical priming site in the sequences immediately upstream of FLO1 (YAR050W). YAL065C is a pseudogene that may encode a homolog of FLO1, and this site was not initially detected by the BLAST search embedded within the primer design process. However, it was detected when a FASTA search was implemented instead. The second instance of mis-priming involved the PCR1 primers for YER158C, which produced a product at half the expected size. This resulted from an 8 bp perfect match to the 3’ end of the forward primer within the expected amplicon. Sequencing confirmed that mis priming had indeed occurred at this alternative priming site, and that this had not been detected by either of the BLAST or FASTA searches carried out during the primer design process.

### Primer libraries

We have designed primers suitable for the deletion of 5941 (90%) of the 6607 ORFs in *S. cerevisiae*. This primer collection is based on the use of URA3 (orotidine-5’-phosphate decarboxylase) selection and 5-fluoroorotic acid (5-FOA) counter-selection procedures [37]. Primer collections using other selectable markers such as LYS5 could also easily be developed.

The tool is applicable to arbitrary genomes. The algorithm employed for the generation of primers is the same for all organisms, and the process differs only by the input data (genome and plasmid/marker sequence), and by the tuning of parameters to allow for the different characteristics of each organism. Therefore the primer design algorithm and the method for gene deletion is generally applicable provided the organism is genetically tractable, the genome sequence is available, and a selection/counterselection mechanism exists. To demonstrate this we developed primer collections for *Schizosaccharomyces pombe* and *Lactococcus lactis* using selectable and counterselectable markers suitable for these organisms.

For *S. pombe* we used URA4 as a selectable marker and FOA for the counter-selection [38, 39] for use in a strain harbouring a URA4 endogenous deletion (ura4-D18) [40]. The use of 5FOA for URA4 counter selection in *S. pombe* often results in a relatively high background of spontaneous 5FOA^*R*^ resistant colonies [41, 42]. Direct repeats present in our deletion cassettes are designed to increase the likelihood FOA^*R*^ colonies arising as a result of recombination. We provide primers for the deletion of 4530 (88%) of the ORFs from *S. pombe*. As with *S. cerevisiae* and *L. lactis* confirmation primers were generated to permit rapid PCR determination of correct homologous integration of the selectable marker and it’s subsequent recovery. We are currently working the design of primers that use a 2 gene selectable marker (ura5+ lys7+) to aid in the selection of lysine auxotrophs and 5FOA resistant colonies arising from marker gene loss through recombination and not spontaneous mutation [42].

The primer collection for *L. lactis* is based on the use of erythromycin/*oroP*/5-fluoroorotate selection and counter selection [43]. We provide primers for 1972 (85%) of the ORFs from *L. lactis*, demonstrating that suitable primers can also be found for prokaryotes. *L. lactis* has many homopolymeric runs in its genome, which required slight refinement of the algorithm for searching for non-specific binding. This refinement, although not necessary for *S. cerevisiae* and *S. pombe*, is still useful for these organisms and as more organisms are investigated in the future, new knowledge about primer design can be incrementally added to the open source software we provide.

Our software is currently unable to generate suitable primers for the deletion of (10.1%) of the ORFs in *S. cerevisae*, (11.9%) of the 5143 ORFs in *S. pombe* and (15.0%) of the 2321 ORFs in *L. lactis*. For these ORFs there were no primers that satisfied all the necessary constraints. For the large majority of these cases we were unable to find a suitable forward primer to pair with any of the potential reverse primer candidates for PCR1. The forward primer is constrained to have a similar length and GC content as the reverse primer.

By relaxing these constraints (for example to allow a wider range of G + C content, or to allow more possibility of dimer formation) it is possible to generate more candidates for testing, potentially increasing the number of ORFs that could potentially be deleted.

All relevant data are available within the paper and its Supporting Information files. Specifically, S1 Primer Sequences. The S1 Primer Sequences is a CSV file containing all primer sequences referred to in the manuscript. Readers may find the following link helpful in interpreting the output: http://www.aber.ac.uk/en/cs/research/cb/projects/precise-deletion/ where all primer sequences can be accessed via a user-friendly web interface, or via a machine-friendly REST-based web service, or downloaded as a MySQL database.

## Discussion

### Method limitations

From the 96 test ORFs deleted, in a number of cases there was an unexpected lack of visible product. In 13 out of 96 cases PCR1 primer pairs failed to yield a visible amplicon at the standard annealing temperature (58°C), but produced the correct product when the annealing temperature was reduced. Temperature gradients showed their optimal temperatures to be less than 58°C. However, a lower annealing temperature should not be used for all ORFs since stringency is reduced. Two of the 96 PCR2 reactions failed to generate a visible amplicon (YLR091W and YER115C). Lower temperature annealing may alleviate this potential problem, but we have not explored this.

In 26 cases there was no visible product in PCR3 and in 10 cases this correlated with the absence of a visible product in either PCR1 or PCR2. In the remaining 16 cases we presume that the absence of a visible PCR3 product resulted from a weak or failed SOEing reaction. Interestingly, when employed to transform *S. cerevisiae*, most of the PCR3 reactions lacking visible products nevertheless yielded colonies on the selective medium, indicating that sufficient product was in fact present to permit inactivation of the target gene. This is a testimony to the efficiency of the optimised transformation protocol, and to the high homologous recombination rate in *S. cerevisiae*. In addition, some of the reactions that gave no visible product in PCR1 nevertheless yielded the expected product in PCR3. Evidently, the lack of a visible product in either PCR1 or PCR3 by no means precluded a successful final outcome.

### The need for new primer libraries

Both *S. cerevisiae* and *S. pombe* have existing single gene deletion collections [45, 46] that are widely used by the research community. However, the code employed to generate the primers required for creating these mutant libraries is no longer available for use. Therefore, if the primers reported do not suit a particular research requirement, new primers have to be designed manually *ab initio*. One motivation for our work was to improve this situation by providing a description of the algorithm together with the actual software, which will allow others to make such modifications as they may require in the future. *L. lactis* does not yet have a gene deletion collection, and this method allows researchers to create their own.

### The need for multiple deletions of genomic elements

Although the functions of 75% of the genes of *S. cerevisiae* are either known or have been predicted with various degrees of confidence, at the time of writing there still remain 848 genes (12%) which are functionally uncharacterised (Percentages acquired from SGD [28]). Among these will be genes that show functional redundancy and it will be necessary to construct multiple mutants to elucidate biological function. A laborious research programme is currently underway, aimed at creating all 6.7 million possible double mutants, of which approximately 5.4 million have so far been obtained [7]. Clearly the process of deleting all possible subsets of genes does not scale, as it is exponential. Therefore an automated targeted method is required.

In the future, more ambitious projects, such as definition of the minimal Yeast genome, will require procedures for constructing strains lacking hundreds and probably even thousands of genes. Perhaps the most exciting such application is the sequential deletion of multiple genes to investigate the minimal eukaryotic gene set. This important problem has so far only been addressed using bioinformatics [47–50]. If it were amenable to direct experimental enquiry it would enable a much greater understanding of eukaryote systems biology, and make easier the addition of novel traits using synthetic biology approaches [51]. Minimal genome projects have the potential to enormously enhance our understanding of the fundamentals of biology and to provide ideal synthetic biology platforms. Most current research into minimal genomes has focussed on a bottom up approach based on DNA assembly [52]. The use of our deletion method provides a top down approach to genome reduction. This has significant advantages over the bottom-up approach taken by Craig Venter and others. Four main considerations recommend it. First, it is extremely doubtful that our current knowledge of the eukaryotic cell is sufficient to allow us to design a minimal genome *de novo*. Second, the intermediate stages in the process (and, particularly, a yeast strain with a minimised metabolic network) represent extremely valuable research tools in their own right. Third, the *Mycoplasma mycoides* work actually relied on yeast genetic engineering tools, which are perhaps the most advanced in any organism. Finally, the data arising from the minimisation process will enable us to refine and improve our knowledge of yeast systems biology. In the top down approach as genes are deleted, lethal edits can be “undone” by reverting to a previous working version. Of the ∼4700 dispensable genes in *S. cerevisiae*, considerably fewer will be dispensable in combination. There will certainly be many minimal gene sets possible, which will be dependent on the temporal order of deletion and the environment in which the organism is grown. The number of combinations possible for step-wise deletion is astronomical and the rational selection of combinations is restricted by gaps in our understanding.

## Supporting Information

S1 Fig PCR1 Agarose Gel. This gel demonstrates PCR1 products for the initial 11 ORFs in lanes 1–11 when a 10:1 ratio of primers (forward:reverse) was employed. Other lanes on this gel show PCR2 results, and are discussed later. Although amplification in PCR1 was good when equimolar amounts of the forward and reverse primers were used, the amplification product was sometimes weak or not visible when a 10:1 ratio of primers was employed. Alternative primer ratios might have proven effective, but this was not explored further. For example, when a 10:1 ratio of primers (forward:reverse) was employed in PCR1, only eight of the initial 11 ORFS amplified well. This can be seen in the lanes numbered 1–11. Lanes 2, 3 and 9 show poor amplification.
(PNG)

S2 Fig Equimolar PCR1 Primers. This gel shows the contrasting situation where equimolar amounts of primers are used for PCR1. The expected product was still obtained in PCR3 but sometimes with further amplification of the PCR1 and PCR2 products. Occasionally, even when PCR1 was successful at a 10:1 primer ratio, the PCR3 amplification produced further amplification of PCR1 and PCR2 products, presumably caused by failure of the SOEing reaction during the first 10 cycles of PCR3.
(PNG)

S3 Fig PCR2 Non-Specific Amplification. When testing the primers selected on the basis of these initial criteria, PCR2 failed for the ORF YGR125W and electrophoretic analysis indicated primer dimer formation that had not been predicted. This gel shows the problem for YGR125W and demonstrates that the reverse primer is at fault. The reverse primer is used in lanes 26, 27, 29 and all show far more dimer than the neighbouring lanes that did not have the reverse primer.
(PNG)

S4 Fig YGR125W and YGL202W. Replacement PCR2 primers were designed for YGR125W and YGL202W using the new improved method, and they gave the expected PCR2 product without apparent dimer production.
(PNG)

S5 Fig DNA Heteroduplex. In addition to products of the expected size (approx 1.25 kb), PCR2 gave an additional product approximately double the expected size (approx 2.5 kb). This additional product was seen on several gels, including the two given here.
(PDF)

S6 Fig YMR272C. Tests on PCR2 for YMR272C to vary the extension time showed that the larger product was still evident despite reduced extension time.
(PNG)

S7 Fig YMR272C Testing. Tests on PCR2 for YMR272C excluding the primers (including just the forward primer (F), including just the reverse primer (R), including both primers (F + R), including no primers (NP) showed that both primers were needed to produce any product.
(PNG)

S8 Fig YHR163W, YPR073C, YGR125W, YJL134W. Confirmation gel for strains YHR163W, YPR073C, YGR125W, YJL134W after selection on FOA, with expected product sizes shown at bottom of lanes.
(PNG)

S9 Fig YGR125W and YNL169C. Confirmation gel for strains YGR125W and YNL169C after selection on FOA, with expected product sizes shown at top of lanes.
(PNG)

S10 Fig PCR1—96 Test ORFs. Gel images showing PCR1 products for the 96 ORFs.
(PDF)

S11 Fig PCR2—96 Test ORFs. Gel images showing PCR2 products for all except 8 of the 96 ORFs. The remaining 8 are shown in the gel in S12 Fig.
(PDF)

S12 Fig PCR2 and PCR3—96 Test ORFs. The remaining eight of the PCR2 results are shown in the first 8 non-marker lanes of this gel, which then also contains the first 24 results for PCR3.
(PNG)

S13 Fig PCR3—96 Test ORFs. Gel images showing the remaining PCR3 products for the 96 ORFs.
(PDF)

S14 Fig Gradient PCR1. Temperature gradients to investigate PCR1 failures for YLR091W, YML097C, YAL058W and YCL050C.
(PNG)

S15 Fig Gradient PCR1. Temperature gradients to investigate PCR1 failures for YDR071C and YHR207C.
(PNG)

S16 Fig Gradient PCR1. Temperature gradients to investigate PCR1 failures for YJL128C, YML088W, YMR018W and YMR278W.
(PNG)

S17 Fig Gradient PCR1. Temperature gradients to investigate PCR1 failures for YBR255W, YAR002W, YIL148W and YJR073C.
(PNG)

S18 Fig Gradient PCR1. Temperature gradients to investigate PCR1 failures for YPL033C.
(PNG)

S1 Table 11 ORFs Products. Table of expected sizes of the products produced for the 11 ORFS.
(CSV)

S2 Table Primer Table for 11 ORFs. Table of primer sequences for the 11 ORFs.
(CSV)

S3 Table YGL202W and YGR125W PCR2. Further forward primers were then chosen for PCR2 for YGL202W and YGR125W.
(CSV)

S4 Table Confirmation Primer Products. Expected product sizes using the confirmation primers. The four resultant strains were verified using confirmation primers from the EuroFAN project (http://www-sequence.stanford.edu/group/yeast_deletion_project/Deletion_primers_PCR_sizes.txt).
(CSV)

S5 Table Summary of the 96 Test ORFs. Table of properties of the PCR results for the 96 ORFs.
(CSV)

S6 Table Primer GC Content. Results of investigation into overall GC content. a Where there is no comment, the expected amplicon was obtained using the standard annealing temperature of 58°C (See Methods). b Only 6 of the 21 bases at the 3’ end of the forward primer are G or C. In general, reactions involving primers with an overall G + C content below 30% either gave a weak product or no visible product at all. However, this was not always the case. Furthermore, some primer pairs with higher G + C contents also failed to yield a visible product. In spite of the apparent failure of some of these reactions under standard conditions, all primer pairs chosen to test overall G + C content yielded the expected product when the reduced temperature touchdown PCR was employed (touchdown PCR with annealing from 60–55°C for 5 cycles and then 55°C for a further 25 cycles). We do not advocate the use of a lower annealing temperature for all ORFs since stringency is reduced. We also examined the efficacy of amplification in relation to the frequency of G + C residues at the 3’ extremity of primers with an overall G + C content above the 30% threshold (S7 Table). We expected reactions to fail if primers lacked a GC clamp and contained two or less G + C residues among the last 8 at the 3’ end. In fact, some primers of this nature did generate amplicons under standard annealing conditions (58°C) whereas others did not.
(CSV)

S7 Table Primer GC Content. Results of investigation into G + C content of the 8 bases at the 3’ end. a Where there is no comment, the expected amplicon was obtained using the standard annealing temperature of 58°C (See Methods). b This reaction gave a product of unexpected size. See also S6 Table.
(CSV)

S8 Table 96 Test Primers. Table of primer sequences for the 96 ORFs.
(CSV)

S1 Sequence Data Sequence Data. Sequence results for the four strains with confirmed deletions.
(ZIP)

S1 Alignments Alignment Files. Alignments of sequencing results with expected sequence after the deletion.
(ZIP)

S1 Algorithm 96 ORFs Selection Criteria. Summary of reasons/methods for choosing the 96 ORFS.
(PDF)

S1 Primer Sequences Primer Sequences. Compressed CSV file of all primers and products for all ORFs in *S. cerevisiae*, *S. pombe* and *L. lactis*.
(BZ2)
