# Supplementary figures and images for: A Tool for Multiple Targeted Genome Deletions that Is Precise, Scar-Free, and Suitable for Automation
Source: PLoS One. 2015 Dec 2;10(12):e0142494. doi: 10.1371/journal.pone.0142494 (PMC4668057; doi:10.1371/journal.pone.0142494)

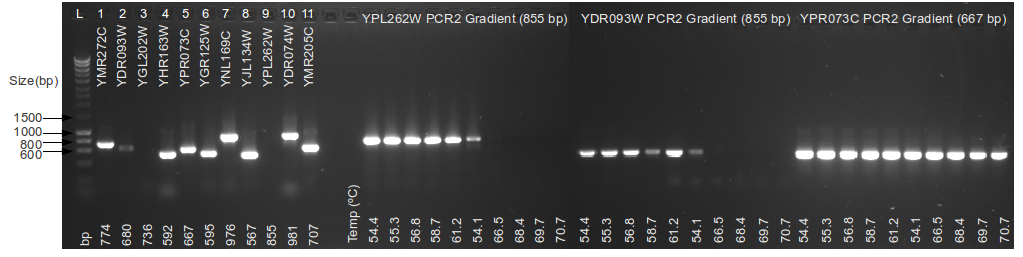

Supplement: S1 Fig — This gel demonstrates PCR1 products for the initial 11 ORFs in lanes 1–11 when a 10:1 ratio of primers (forward:reverse) was employed. Other lanes on this gel show PCR2 results, and are discussed later. Although amplification in PCR1 was good when equimolar amounts of the forward and reverse primers were used, the amplification product was sometimes weak or not visible when a 10:1 ratio of primers was employed. Alternative primer ratios might have proven effective, but this was not explored further. For example, when a 10:1 ratio of primers (forward:reverse) was employed in PCR1, only eight of the initial 11 ORFS amplified well. This can be seen in the lanes numbered 1–11. Lanes 2, 3 and 9 show poor amplification. (PNG) [file pone.0142494.s001.png]

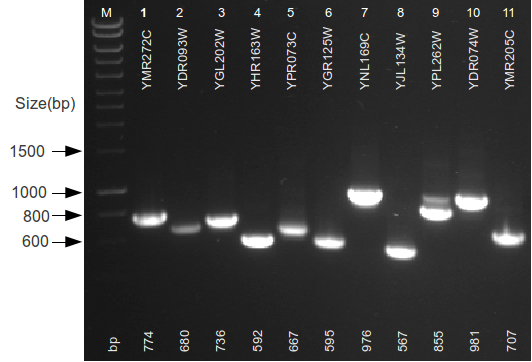

Supplement: S2 Fig — This gel shows the contrasting situation where equimolar amounts of primers are used for PCR1. The expected product was still obtained in PCR3 but sometimes with further amplification of the PCR1 and PCR2 products. Occasionally, even when PCR1 was successful at a 10:1 primer ratio, the PCR3 amplification produced further amplification of PCR1 and PCR2 products, presumably caused by failure of the SOEing reaction during the first 10 cycles of PCR3. (PNG) [file pone.0142494.s002.png]

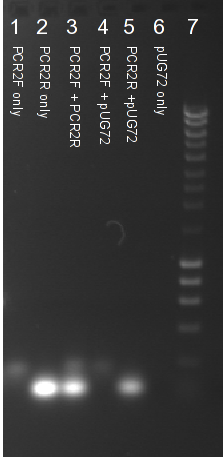

Supplement: S3 Fig — When testing the primers selected on the basis of these initial criteria, PCR2 failed for the ORF YGR125W and electrophoretic analysis indicated primer dimer formation that had not been predicted. This gel shows the problem for YGR125W and demonstrates that the reverse primer is at fault. The reverse primer is used in lanes 26, 27, 29 and all show far more dimer than the neighbouring lanes that did not have the reverse primer. (PNG) [file pone.0142494.s003.png]

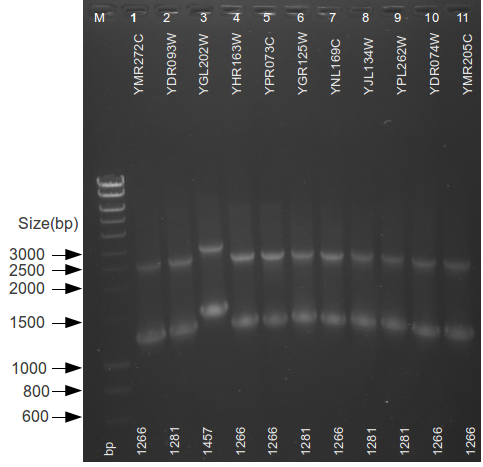

Supplement: S4 Fig — Replacement PCR2 primers were designed for YGR125W and YGL202W using the new improved method, and they gave the expected PCR2 product without apparent dimer production. (PNG) [file pone.0142494.s004.png]

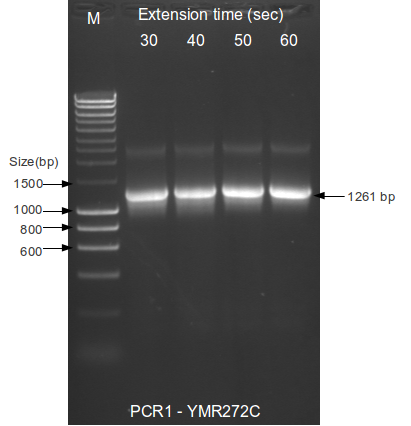

Supplement: S6 Fig — Tests on PCR2 for YMR272C to vary the extension time showed that the larger product was still evident despite reduced extension time. (PNG) [file pone.0142494.s006.png]

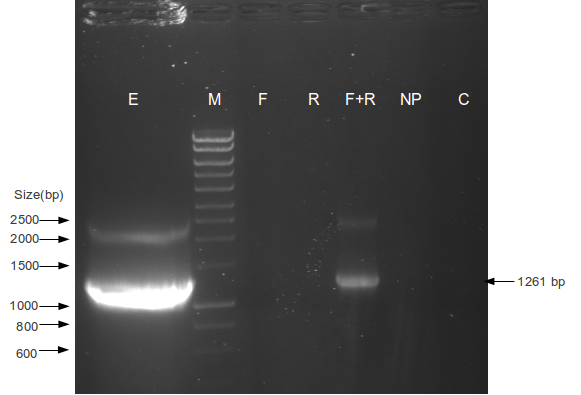

Supplement: S7 Fig — Tests on PCR2 for YMR272C excluding the primers (including just the forward primer (F), including just the reverse primer (R), including both primers (F + R), including no primers (NP) showed that both primers were needed to produce any product. (PNG) [file pone.0142494.s007.png]

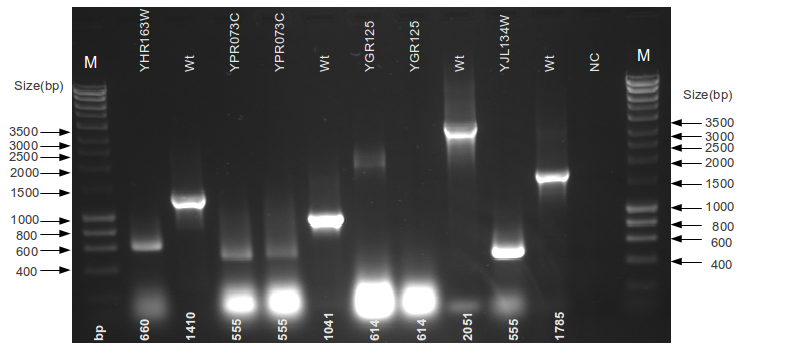

Supplement: S8 Fig — Confirmation gel for strains YHR163W, YPR073C, YGR125W, YJL134W after selection on FOA, with expected product sizes shown at bottom of lanes. (PNG) [file pone.0142494.s008.png]

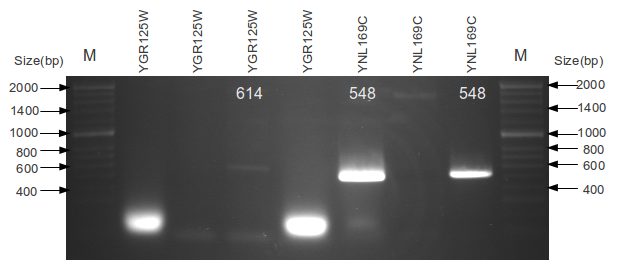

Supplement: S9 Fig — Confirmation gel for strains YGR125W and YNL169C after selection on FOA, with expected product sizes shown at top of lanes. (PNG) [file pone.0142494.s009.png]

S10 Fig A

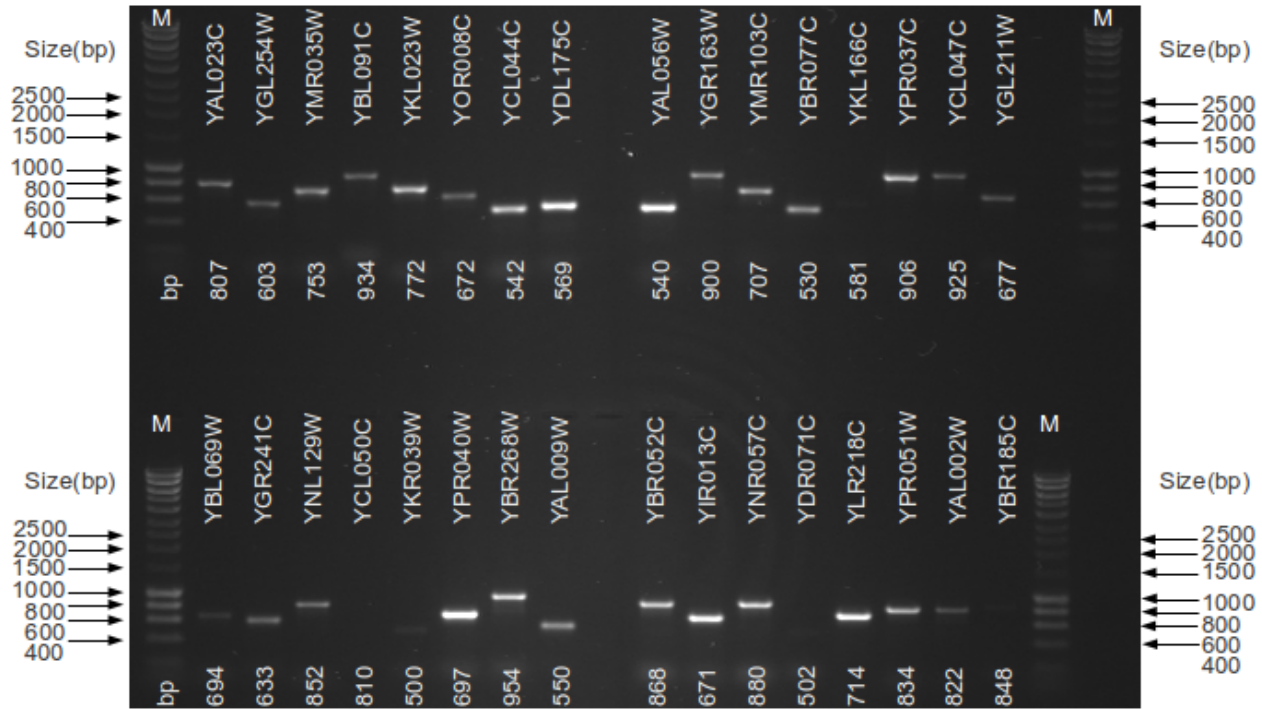

S10 Fig B

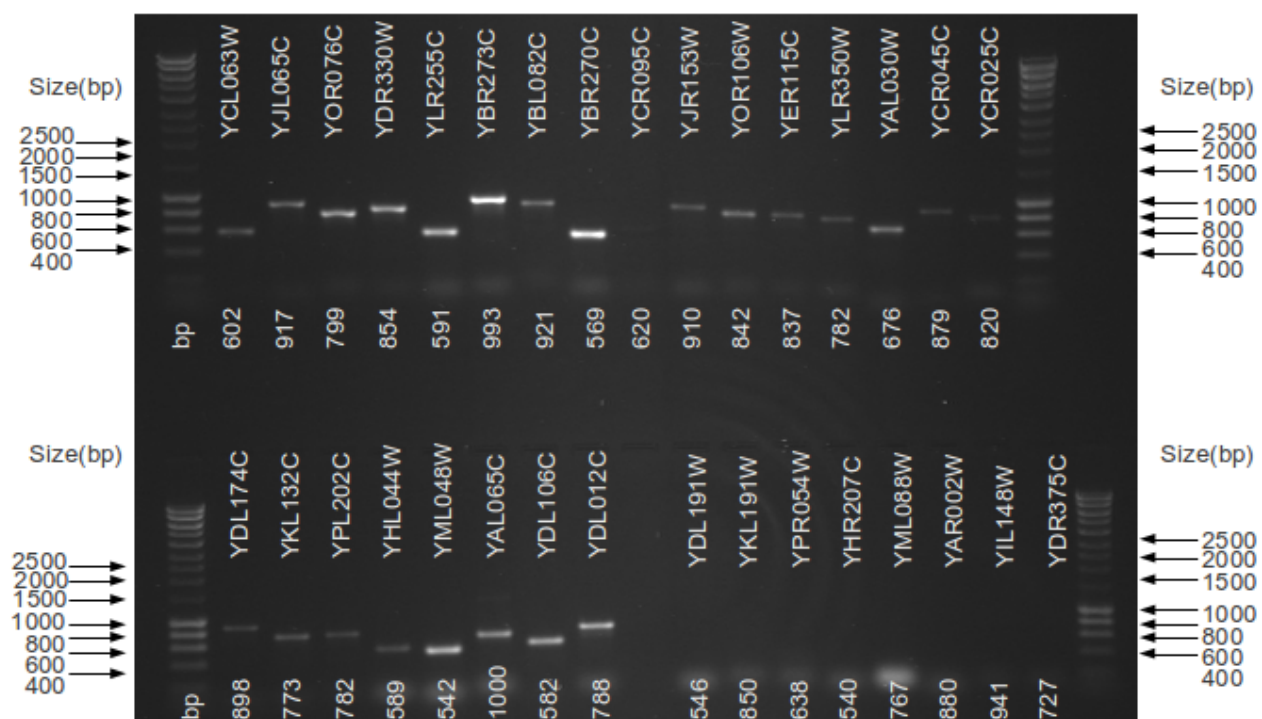

S10 Fig C

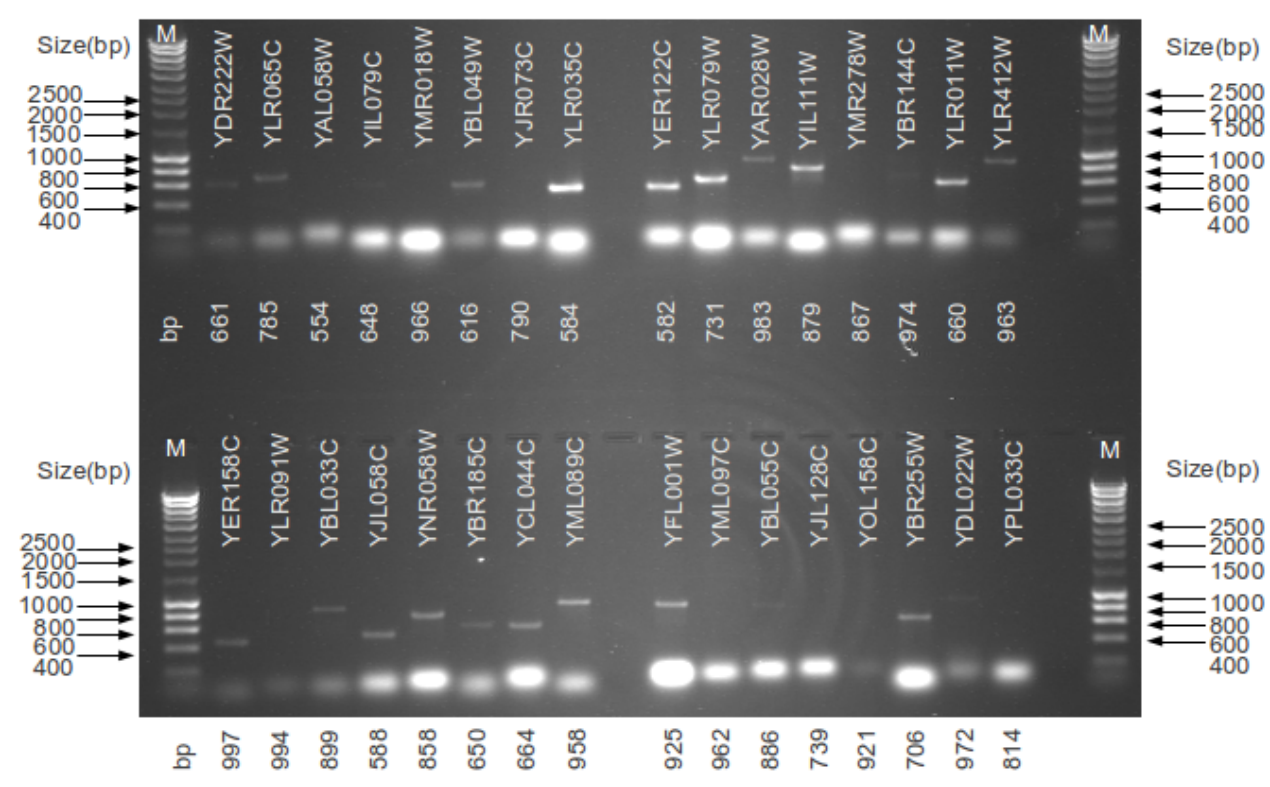

Figure S10 A, B, C. Gel images showing PCR1 products for the 96 ORFs.

Supplement: S10 Fig — Gel images showing PCR1 products for the 96 ORFs. (PDF) [file pone.0142494.s010.pdf]

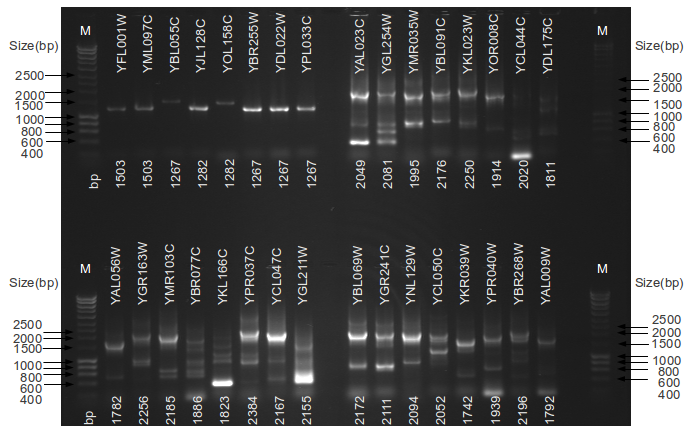

Supplement: S12 Fig — The remaining eight of the PCR2 results are shown in the first 8 non-marker lanes of this gel, which then also contains the first 24 results for PCR3. (PNG) [file pone.0142494.s012.png]

S13 Fig A

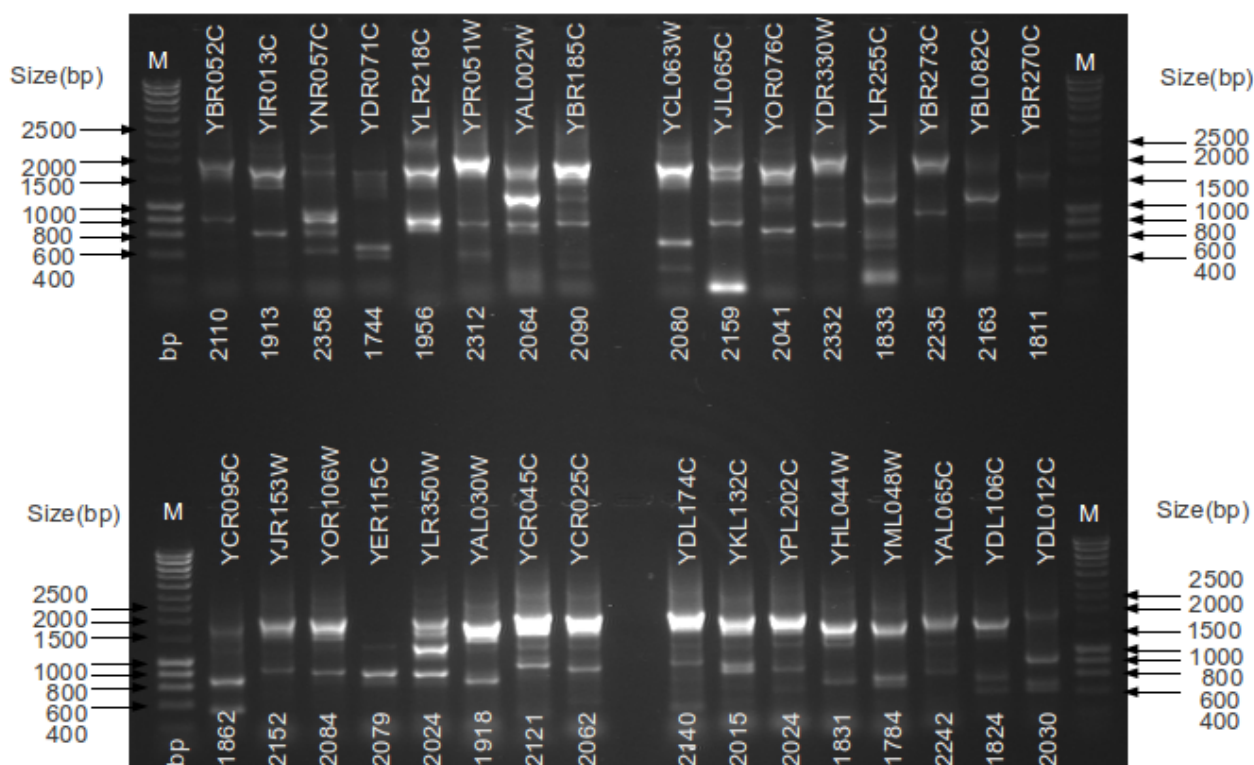

S13 Fig B

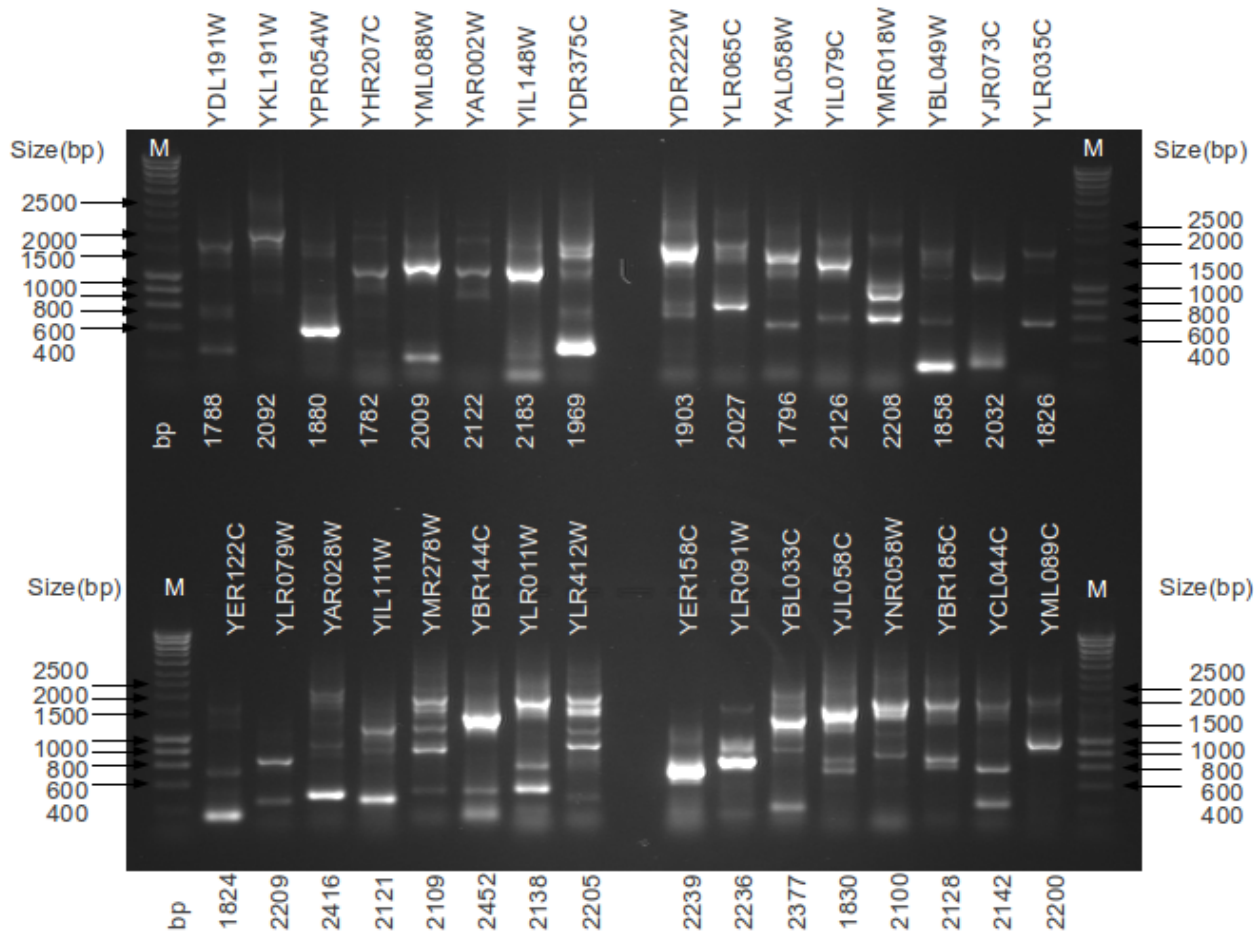

S13 Fig C

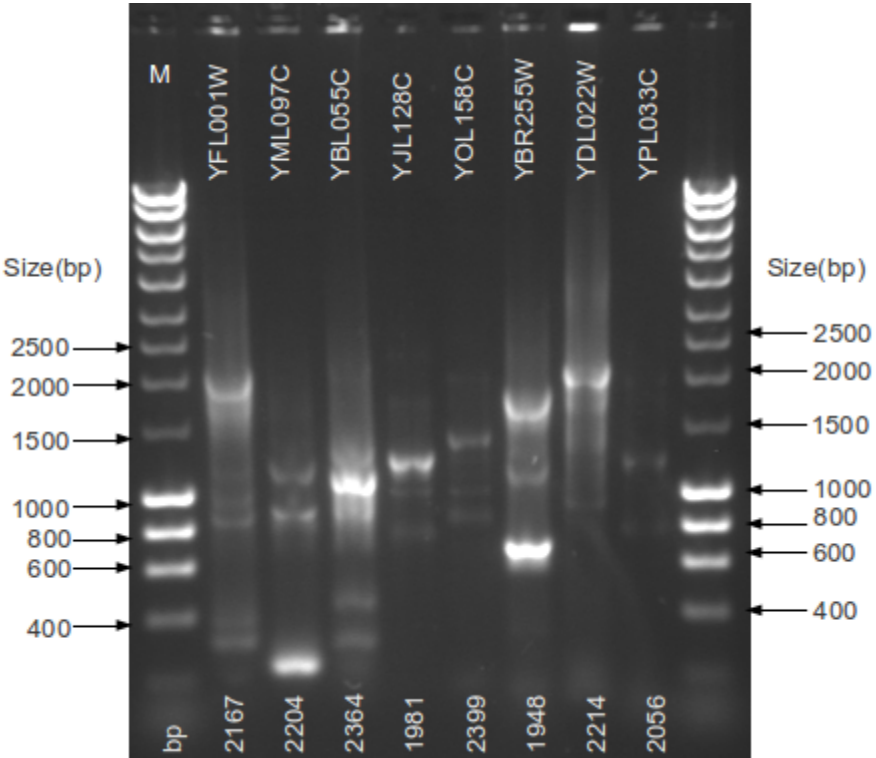

Figure S13 A, B, C. Gel images showing the remaining PCR3 products for the 96 ORFs.

Supplement: S13 Fig — Gel images showing the remaining PCR3 products for the 96 ORFs. (PDF) [file pone.0142494.s013.pdf]

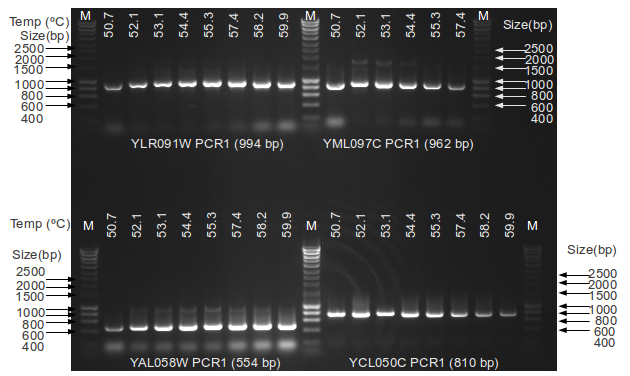

Supplement: S14 Fig — Temperature gradients to investigate PCR1 failures for YLR091W, YML097C, YAL058W and YCL050C. (PNG) [file pone.0142494.s014.png]

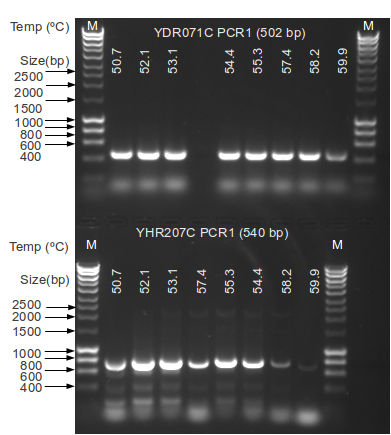

Supplement: S15 Fig — Temperature gradients to investigate PCR1 failures for YDR071C and YHR207C. (PNG) [file pone.0142494.s015.png]

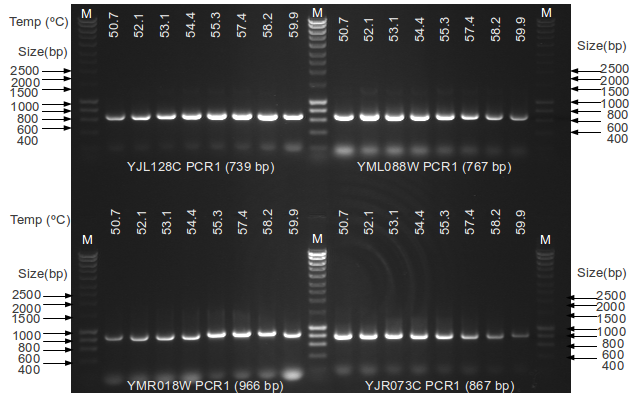

Supplement: S16 Fig — Temperature gradients to investigate PCR1 failures for YJL128C, YML088W, YMR018W and YMR278W. (PNG) [file pone.0142494.s016.png]

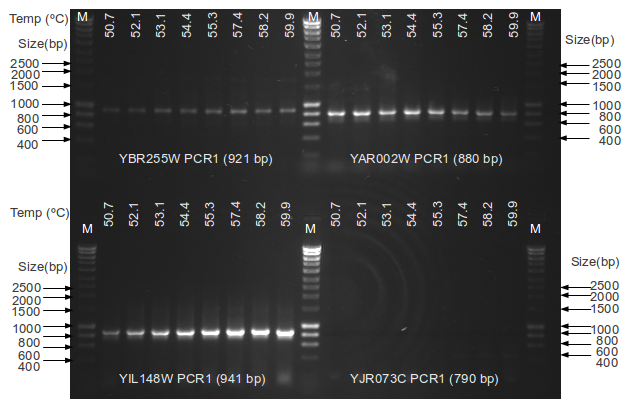

Supplement: S17 Fig — Temperature gradients to investigate PCR1 failures for YBR255W, YAR002W, YIL148W and YJR073C. (PNG) [file pone.0142494.s017.png]

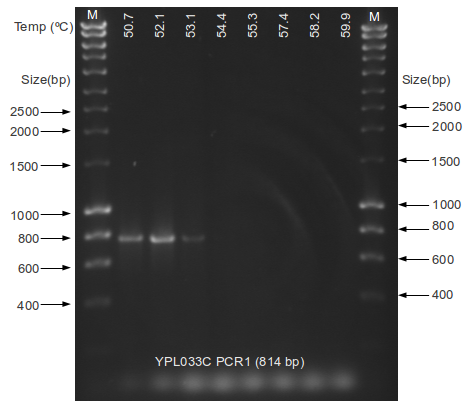

Supplement: S18 Fig — Temperature gradients to investigate PCR1 failures for YPL033C. (PNG) [file pone.0142494.s018.png]
