# Supplementary material for: A Tool for Multiple Targeted Genome Deletions that Is Precise, Scar-Free, and Suitable for Automation
Source: PLoS One. 2015 Dec 2;10(12):e0142494. doi: 10.1371/journal.pone.0142494 (PMC4668057; doi:10.1371/journal.pone.0142494)
Supplement: S5 Fig — In addition to products of the expected size (approx 1.25 kb), PCR2 gave an additional product approximately double the expected size (approx 2.5 kb). This additional product was seen on several gels, including the two given here. (PDF) [file pone.0142494.s005.pdf]

S5 Fig A

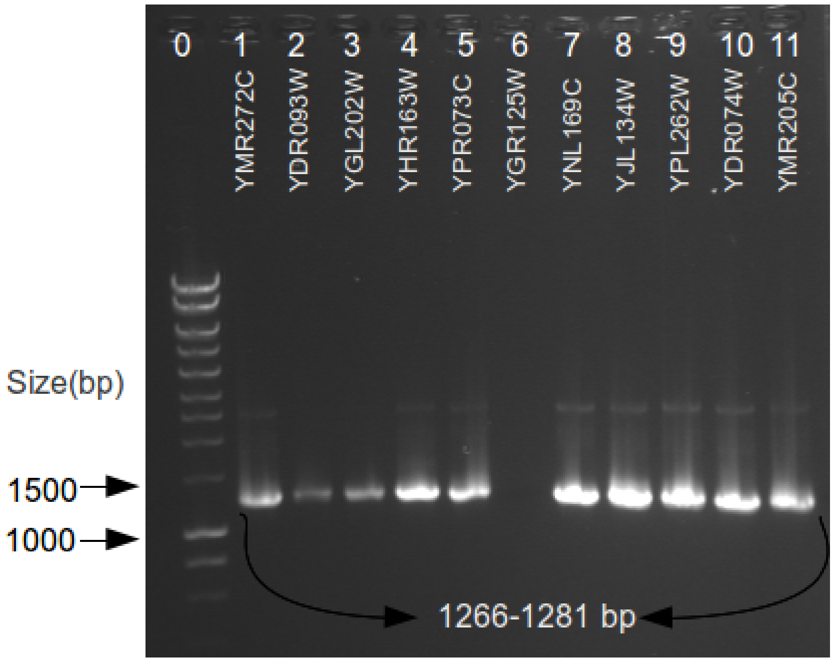

S5 Fig B

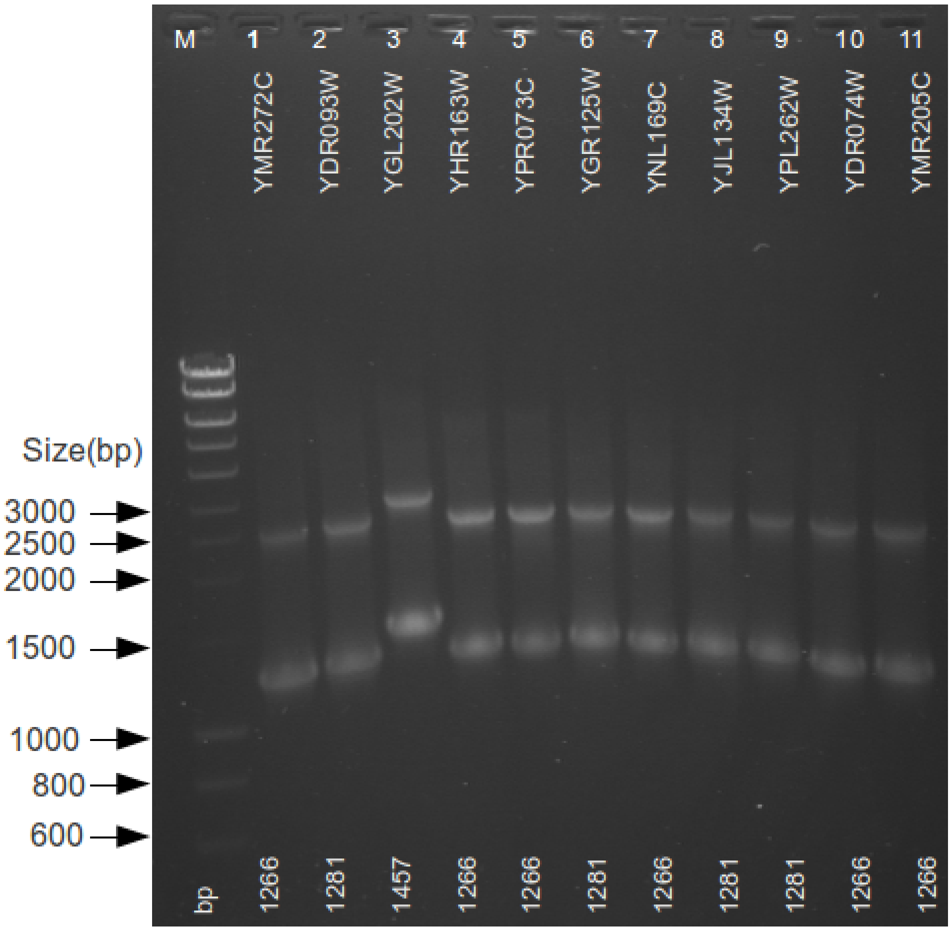

Figure S5 A and B. In addition to products of the expected size (approx 1.25 kb), PCR2 gave an additional product approximately double the expected size (approx 2.5 kb). This additional product was seen on several gels, including the two given here.
