# Supplementary material for: A Tool for Multiple Targeted Genome Deletions that Is Precise, Scar-Free, and Suitable for Automation
Source: PLoS One. 2015 Dec 2;10(12):e0142494. doi: 10.1371/journal.pone.0142494 (PMC4668057; doi:10.1371/journal.pone.0142494)
Supplement: S11 Fig — Gel images showing PCR2 products for all except 8 of the 96 ORFs. The remaining 8 are shown in the gel in S12 Fig. (PDF) [file pone.0142494.s011.pdf]

S11 Fig A

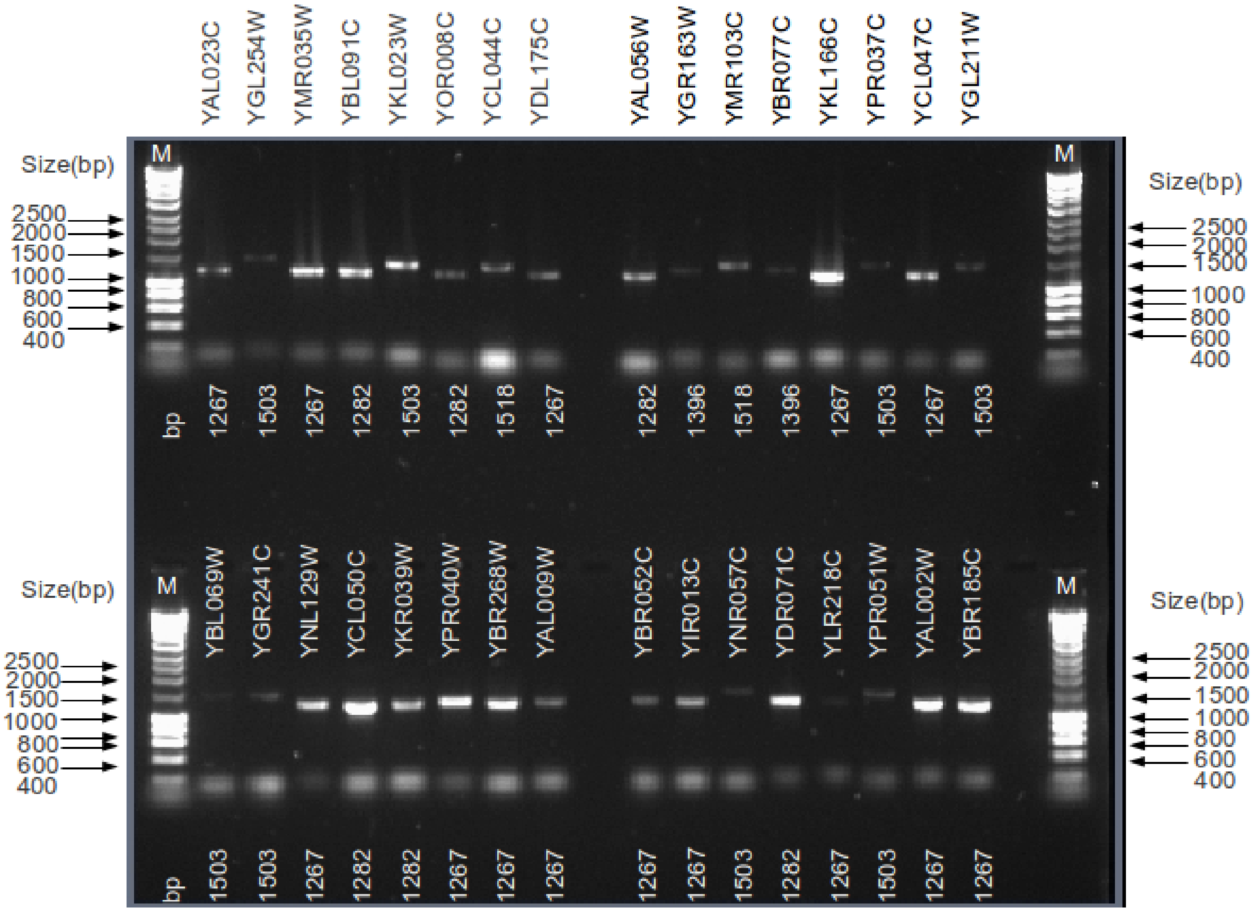

S11 Fig B

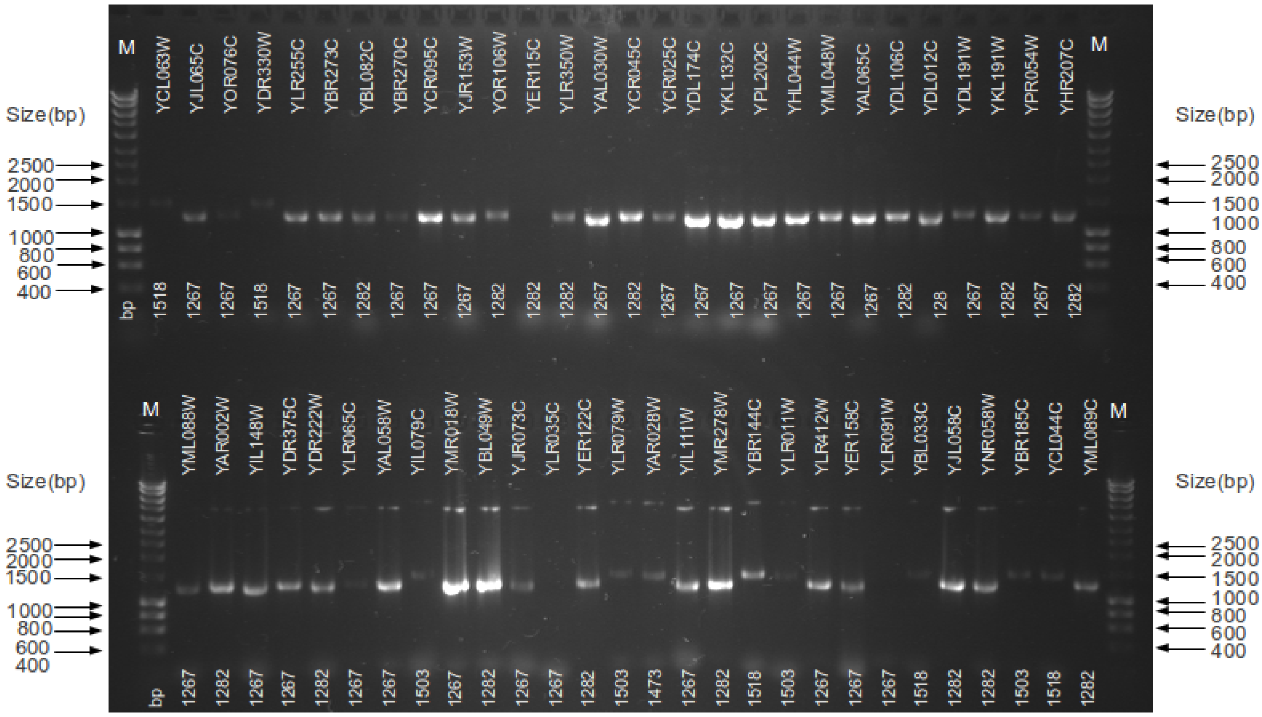

Figure S11 A, B. Gel images showing PCR2 products for all except 8 of the 96 ORFs. The remaining 8 are shown in the gel in Figure S12.
