# Supplementary material for: A Tool for Multiple Targeted Genome Deletions that Is Precise, Scar-Free, and Suitable for Automation
Source: PLoS One. 2015 Dec 2;10(12):e0142494. doi: 10.1371/journal.pone.0142494 (PMC4668057; doi:10.1371/journal.pone.0142494)
Supplement: S1 Algorithm — Summary of reasons/methods for choosing the 96 ORFS. (PDF) [file pone.0142494.s029.pdf]

Summary of reasons/methods for choosing the 96 ORFs.

Choices of ORFs were determined by the following:

- 32 ORFS that were predicted to have good primers under every condition
- 32 ORFS to test the new dimerism scoring method. These should include a good range of predicted outcomes, including primers that were predicted to fail because of dimer. These 32 ORFs shouls have primers that are good in all other respects
- 32 ORFS to test the effect GC content (16 to test the effect of overall average GC content, and 16 to test the effect of GC content in the 8 bases at the 3' end)

The 32 ORFS chosen to have good primers:

- have primers
- are non-essential as given by the MIPS viability catalog or by the collection notes that came with our Eurofan collection of single gene deletants.
- have primers with:
    - no unwanted non-specific binding and low hairpin, dimer and self dimer scores (<= 10)
    - between 40% to 50% GC content overall
    - at least 37% GC content in 8 bases at 3' end for both primers (at least 3/8 at 3' end)
- have confirmation primers and these make products of less than 3000 bases

The 32 ORFS chosen to test dimerism scoring:

- have primers
- are non-essential as given by the MIPS viability catalog or by the collection notes that came with our Eurofan collection of single gene deletants.
- have primers with:
    - between 40% to 50% GC content overall
    - at least 37% GC content in 8 bases at 3' end for both primers (at least 3/8 at 3' end)
- have confirmation primers and these make products of less than 3000 bases
- have no unwanted non-specific binding
- have a range of dimer and self dimer scores

The 32 ORFS chosen to test effect of GC content:

- have primers
- are non-essential as given by the MIPS viability catalog or by the collection notes that came with our Eurofan collection of single gene deletants.
- have primers with:
    - no unwanted non-specific binding and low hairpin, dimer and self dimer scores (<= 10)
- have confirmation primers and these make products of less than 3000 bases
- demonstrate a range of GC content from lowest to highest with half (16 ORFs) to test overall GC content range and half (16 ORFs) to test 3' end GC content
- The resulting choices, showing the overall GC percentage, the number of GCs in the last 8 bases and the result of an 8 base sliding window average across the length of the primer are given in GCExptsByOverall.txt and GCExptsByEnd.txt.
